# Safety and efficacy of cryoablation for atrial fibrillation in young patients: A multicenter experience in the 1STOP project

**DOI:** 10.1002/clc.23951

**Published:** 2022-11-29

**Authors:** Emanuele Bertaglia, Saverio Iacopino, Roberto Verlato, Giuseppe Arena, Paolo Pieragnoli, Claudio Tondo, Giulio Molon, Massimiliano Manfrin, Giovanni B. Perego, Giovanni Rovaris, Francesco Rivezzi, Massimo Mantica, Umberto Startari, Luigi Sciarra

**Affiliations:** ^1^ Department of Cardiac, Thoracic and Vascular Sciences and Public Health University of Padua Padua Italy; ^2^ Maria Cecilia Hospital GVM Care & Research Cotignola Italy; ^3^ ULSS 6 Euganea Ospedale di Camposampiero Camposampiero Italy; ^4^ Ospedale delle Apuane Massa Italy; ^5^ Ospedale Careggi University of Florence Firenze Italy; ^6^ Heart Rhythm Center, Department of Clinical Electrophysiology&Cardiac Pacing Monzino Cardiac Center, IRCCS Department of Biochemical Surgical and Dentist Sciences University of Milan Milan Italy; ^7^ IRCCS Sacro Cuore don Calabria Negrar Italy; ^8^ Ospedale Bolzano Italy; ^9^ Ospedale San Luca, Istituto Auxologico Milano Italy; ^10^ ASST San Gerardo di Monza Monza Italy; ^11^ Istituto Sant'Ambrogio Milano Italy; ^12^ Fondazione Toscana Gabriele Monasterio Pisa Italy; ^13^ Policlinico Casilino Roma Italy

**Keywords:** atrial fibrillation, catheter ablation, cryoballoon ablation, young patients

## Abstract

**Background:**

Atrial fibrillation (AF) is an uncommon arrhythmia in young adults without structural heart disease, and cryoballoon pulmonary vein isolation (CB‐PVI) is an important therapeutic strategy for rhythm control in patients with drug‐refractory AF. The aim of this analysis was to evaluate efficacy and safety of CB‐PVI in a large cohort of young patients in comparison with middle‐aged adults in a real‐world setting.

**Methods:**

From 2012 to 2020, a total of 3033 patients with AF underwent CB‐PVI and were followed prospectively in the framework of the 1STOP Clinical Service project, involving 34 Italian centers. Out of 3033 total 1STOP project subjects, a subgroup of 1318 patients were defined which included a YOUNG group (age ≤ 45 years; *n* = 368) and a MIDDLE‐AGED group (age 60–65 years; *n* = 950).

**Results:**

The acute success rate of PVI did not differ between the two cohorts (99.9 ± 1.3% vs. 99.8 ± 3.2%, *p* = 0.415). There was no difference in procedural characteristics, and periprocedural complication rates were similar among the two cohort (1.9% vs. 2.3%, *p* = 0.646). The 12‐month freedom from AF recurrence was 88.9% (95% confidence interval [CI]: 84.7–92.0) in the YOUNG cohort and 85.6% (95% CI: 82.9–88.0) in the MIDDLE‐AGED group. At 36‐month follow‐up, freedom from AF recurrence was 72.4% (65.5%–78.2%) and 71.8% (67.7%–75.6%), respectively with no significant difference among groups (*p* = 0.550).

**Conclusion:**

CB‐PVI had similar efficacy and safety in YOUNG and MIDDLE‐AGED patients. Younger age did not affect acute procedural results, complication rate, or AF recurrence after a single procedure.

## WHAT'S NEW?

- This is the first multicenter analysis to evaluate the differences in efficacy and safety of a single ablation technique (cryoballoon pulmonary veins isolation) for atrial fibrillation between young and middle‐aged patients.
- Our analysis demonstrated the same rate of acute success, freedom from recurrent atrial fibrillation between young and middle‐aged patients, despite (as expected) older patients having more comorbidities.
- Safety outcomes of the procedure did not differ between young and middle‐aged patients, with a very low complication rate overall in both cohorts.
- The rate of repeat ablations following the index procedure was higher in young as compared to middle‐aged

## 1 INTRODUCTION

Atrial fibrillation (AF) is the most common sustained arrhythmia in clinical practice, affecting approximately 5.5% of the adult population in Western countries.^1^ Patients affected with AF are at high risk for cardiovascular events, stroke, dementia, and death.

Pulmonary veins isolation (PVI), by means of either radiofrequency (RF) or cryoballoon (CB) catheter ablation, has emerged as a cornerstone strategy in the management of symptoms and rhythm control in drug‐refractory AF patients,^2^ and the efficacy and safety of CB‐PVI resulted comparable to point‐by‐point RF‐PVI, in a prospective randomized trial.^3^

Despite age being one of the strongest risk factors for predicting AF, the prevalence of AF‐onset in young patients is rising. Compared with older patients, young patients have lower comorbidities, a more symptomatic clinical course, and an aversion toward life‐long medication intake.^4^, ^5^ In the absence of specific literature, the management of young patients with AF has been mostly extrapolated from trials of older patients. To date, little is known about the age‐dependent differences regarding the outcomes of CB‐PVI, and there is a lack of data investigating the longer‐term follow‐up of young patients. Consequently, only a few studies have investigated the clinical outcomes of catheter ablation in young patients.^6^, ^7^, ^8^, ^9^, ^10^, ^11^ Thus, the aim of this analysis was to prospectively compare efficacy and safety of CB‐PVI for patients with AF between a cohort of young (≤45 years) and middle‐aged (60–65 years) patients and to evaluate the predictors of AF recurrence after CB‐PVI in a multicenter Italian clinical Project.

## 2 METHODS

### 2.1 Study population

From April 2012 to August 2020, 3033 patients with paroxysmal or persistent AF who had undergone CB‐PVI (Arctic Front or Arctic Front Advance; Medtronic, Inc.) in 34 Italian institutions were prospectively followed, according to each center's clinical practice, by means of in‐hospital visits through the framework of the of One Shot to PVI (1STOP) project. Patients with previous AF ablations and lost to follow‐up data were excluded. In this sub‐group analysis, 1318 patients were studied and divided, into two groups according to their age, including: ≤45 years (YOUNG *n* = 368) and between 60 and 65 years (MIDDLE‐AGED *n* = 950). The age range 60–65 years was chosen for middle‐aged patients as this age group is the most represented in the 1STOP project population.

### 2.2 Cryoablation procedure

Patients were treated under general anesthesia or conscious sedation. A transseptal needle puncture for left atrial (LA) access was immediately followed by a heparin bolus delivery. The subsequent heparin delivery was titrated to maintain an activated clotting time of above 300 s. A dedicated delivery sheath (FlexCath or FlexCath Advance; Medtronic, Inc.) was used to handle the balloon catheter and/or guidewire assembly during the ablation procedure. Ablations were performed with a 23‐ and/or 28‐mm CB, which was advanced by an over‐a‐wire method into the LA. The number and length of freeze applications, as well as postablation testing methods varied according to each center's practice. During freezing of the right‐sided PVs, phrenic nerve function was monitored with continuous pacing maneuvers to record diaphragmatic performance. Acute PVI was consistently defined as electrical conduction isolation confirmed by bidirectional block and assessed using the dedicated diagnostic mapping catheter (Achieve or Achieve Advance mapping catheter; Medtronic, Inc.) and/or a standard circular diagnostic mapping catheter. Acute procedural success was defined as the ratio between the number of effectively isolated PVs and the number of target PVs.

### 2.3 Data collection, follow‐up, and reporting

At the baseline visit, clinical patient characteristics were collected, including: age, gender, body mass index, AF‐related symptoms, EHRA score, cardiovascular risk factors, previous history of cardiovascular and thromboembolic events, thromboembolic risk index (i.e., CHA_2_DS_2_‐VASc), and echocardiographic parameters. During the index CB ablation procedure, further data were recorded within the procedural hospitalization, including data on anticoagulation strategy, ablation and procedural durations, and fluoroscopic radiation exposure. Routine follow‐up assessments were made in accordance with the standard clinical practices at each center through in‐hospital visits or telephone interviews. Typically, follow‐up visits were scheduled for every 3 months after the index ablation during the first 12 months, and for every 6 months thereafter. Each follow‐up examination included an AF‐related symptoms' review, ECG for arrhythmic event assessment, and 24‐h ECG Holter monitoring. Patients were routinely discharged with previously ineffective antiarrhythmic agents that were typically discontinued after 12 weeks if the patient had paroxysmal AF and at 6 months if they had persistent AF. The management of antiarrhythmic agents was left to the discretion of the clinical practice of each center. The first 90‐days after AF ablation were considered a landmark blanking period during which any arrhythmia occurrence would not be counted against the long‐term efficacy, to allow time for cardiac healing following cryoablation.

### 2.4 Endpoint

The primary endpoint of the analysis consisted of recurrence of AF, defined as any episode of AF, atrial tachycardia, or atrial flutter (symptomatic or asymptomatic) lasting ≥30 s in duration after the 90‐day blanking period. Secondary endpoints were repeat ablation(s) after the index procedure. All reported procedural and peri‐procedural complications were recorded, and adverse event classifications of minor or major events were made in accordance with the previously published worldwide survey on AF ablation.^12^

### 2.5 Project design

The 1STOP project is a retrospective examination of CB ablations conducted in 34 Italian cardiology centers. This project is part of a larger Italian medical care research project (One Hospital Clinical Service) that is a clinical data repository for an integrated network consortium of Italian cardiac hospitals with the shared goal of evaluating and improving the usage of medical therapies in clinical practice through the mutual collection, examination, and analysis of pooled data. The project and data collection activities conform to the principles outlined in the Declaration of Helsinki, and each patient included in the One Hospital Clinical Service project provided informed written consent. Detailed methods for follow‐up, data collection, reporting, and data repository have been previous described in detail for the 1STOP project.^13^

### 2.6 Statistical analysis

Descriptive statistics were used to summarize patient characteristics, including: mean, standard deviation, minimum, maximum, and median with the interquartile range (IQR) for continuous variables, and counts and percentages for categorical variables. Comparisons between groups have been performed using Wilcoxon's Test for continuous variables, while comparisons of categorical variables have been performed using *χ* ^2^‐test or Fisher's exact test for extreme proportions, as appropriate. The analyses of time‐to‐first event were described using Kaplan–Meier curves and compared between the groups with the log‐rank test. The annual rates of complications were reported, together with the 95% confidence intervals [CI]. To identify predictors of AF recurrence, a cox regression model was imputed for both univariate and multivariate analyses. Parameters that were identified as significant in univariate analyses (*p* < 0.10) were also analyzed in a multivariate model. Statistical tests were based on a two‐sided significance level of 0.05. The SAS software, version 9.4, (SAS Institute Inc.) was used to perform statistical analyses.

## 3 RESULTS

### 3.1 Baseline characteristics

Demographic, clinical, and echocardiographic characteristics of the 1318 patients are summarized in Table 1. MIDDLE‐AGED patients were more frequently female, had a higher body mass index, and had a higher prevalence of persistent AF. Comorbidities (hypertension, diabetes, and chronic kidney disease) were more frequent in MIDDLE‐AGED than in YOUNG patients. Also, older patients had larger LA diameter, larger LA indexed volume, and lower left ventricular ejection fraction. MIDDLE‐AGED patients were more frequently on antiarrhythmic drugs (AAD): there was no difference in class I AAD use before procedure, meanwhile class III AAD were more frequently used in MIDDLE‐AGED patients.

**Table 1:** Baseline characteristics of the study population

Baseline characteristics	Total (*n* = 1318)	≤45 years (*n* = 368)	60–65 years (*n* = 950)	*p*‐Value
Age at first ablation (years)	55.8 ± 11.4	38.4 ± 6.2	62.6 ± 1.7	<0.001
Female sex	24.7% (325)	12.8% (47)	29.3% (278)	<0.001
BMI	26.7 ± 4.3	26.2 ± 4.4	26.9 ± 4.2	<0.001
Any relevant arrhythmia symptoms	80.4% (1060)	80.4% (296)	80.4% (764)	0.996
Type of Atrial fibrillation				<0.001
Paroxysmal	77.2% (1018)	87.0% (320)	73.5% (698)	
Persistent	22.8% (300)	13.0% (48)	26.5% (252)	<0.001
History of flutter	16.5% (217)	11.9% (44)	18.4% (173)	0.011
Months from first atrial tachyarrhythmia episode	51.5 ± 64.7	47.0 ± 54.5	53.3 ± 68.3	
NYHA ≥2 b	15.1% (199)	10.4% (39)	16.9% (160)	0.025
History of stroke/TIA	4.0% (52)	1.5% (5/340)	5.0% (47)	0.005
Hypertension	43.3% (570)	15.8% (58)	54.1% (512)	<0.001
CHA₂DS₂‐VASc^a^				
0	34.6% (457)	68.7% (256)	20.8% (201)	<0.001
1	39.6% (521)	27.9% (103)	44.3% (418)	
2	17.3% (228)	2.4% (8)	23.3% (220)	
3	6.3% (83)	0.3% (1)	8.7% (82)	
4	1.4% (18)	0.3% (1)	1.8% (17)	
≥5	0.9% (11)	0.3% (1)	1.1% (10)	
Diabetes	6.7% (89)	2.3% (8)	8.5% (81)	<0.001
Chronic kidney disease	1.7% (23)	0.0% (0/319)	2.4% (23)	0.005
Echocardiography measurements				
LVEF (%)	59.2 ± 6.8	59.7 ± 7.2	59.0 ± 6.6	0.008
LVEDV (ml)	121.1 ± 53.6	124.1 ± 52.3	119.6 ± 54.3	0.450
LA Diameter (mm)	41.5 ± 7.4	38.6 ± 7.1	42.8 ± 7.2	<0.001
Left atrial volume indexed (cm^3^/m^2^)	36.4 ± 15.2	29.8 ± 10.6	38.9 ± 15.9	<0.001
AAD therapy				
Class I AAD	41.8% (550)	43.3% (159)	41.2% (391)	0.502
Class III AAD	31.5% (415)	19.7% (72)	36.1% (343)	<0.001
Class I or III AAD	66.2% (872)	58.0% (213)	69.5% (659)	<0.001
Non‐AAD therapy				
Thrombin inhibitor	20.9% (276)	10.9% (40)	24.8% (236)	<0.001
Factor XA inhibitor	21.0% (277)	14.9% (55)	23.4% (222)	<0.001
Only Antiplatelet	5.1% (67)	3.9% (14)	5.6% (53)	0.244
OAC + Antiplatelet	1.9% (25)	1.2% (4)	2.2% (21)	0.260
No Antiplatelet/OAC therapies	14.6% (192)	32.9% (121)	7.5% (71)	<0.001
Beta‐blockers	53.1% (700)	39.3% (146)	58.3% (554)	<0.001
Ace‐inhibitors	32.9% (433)	13.6% (50)	40.3% (383)	<0.001
Diuretics	13.5% (177)	3.9% (12)	17.3% (165)	<0.001

*Note*: Continuous variables are expressed as mean ± SD. Categorical variables are expressed as no. (%).

Abbreviations: AAD, antiarrhythmic drug; BMI, body mass index; CKD, chronic kidney disease; LVEDV, left ventricular end‐diastolic volume; LVEF, left ventricular ejection fraction; OAC, oral anti‐coagulant; TIA, transient ischemic attack

^a^ CHA2DS2‐VASc score risk ranges from 0 to 9, with higher scores indicating a greater risk of stroke.

^b^ NYHA New York Heart Association Classification for heart failure ranges from class I to IV, classifies patients in one of four categories based on their limitations during physical activity.

### 3.2 Acute procedural result

Data detailing acute results are summarized in Table 2. The acute success rate was 99.8 ± 2.8% for the entire population, with no statistical difference between YOUNG and MIDDLE‐AGED groups (99.9 ± 1.3% vs. 99.8 ± 3.2%, *p* = 0.415), respectively. There was no difference in overall procedure duration (100.8 ± 44.6 vs. 99.0 ± 43.9, *p* = 0.468), fluoroscopy duration (25.7 ± 14.7 vs. 25.2 ± 14.8, *p* = 0.560) and ablation time (26.5 ± 55.3 vs. 25.0 ± 47.6, *p* = 0.075) between the two groups, respectively. At the end of procedure sinus rhythm was obtained in 1275 (96.8%) patients, with no difference between YOUNG and MIDDLE‐AGED groups (359 [97.4%] vs. 916 [96.5%], *p* = 0.894), respectively.

**Table 2:** Procedural characteristics

Procedural characteristics	Total (*n* = 1318)	≤45 years (*n* = 368)	60–65 years (*n* = 950)	*p*‐Value
Procedure duration (min)	99.5 ± 44.1	100.8 ± 44.6	99.0 ± 43.9	0.468
Fluoroscopy duration (min)	25.3 ± 14.8	25.7 ± 14.7	25.2 ± 14.8	0.560
Ablation time (min)	25.4 ± 49.9	26.5 ± 55.3	25.0 ± 47.6	0.075
Acute success rate	99.8 ± 2.8	99.9 ± 1.3	99.8 ± 3.2	0.415
Preablation sinus rhythm	74.6% (943/1264)	80.8% (291/360)	72.1% (652/904)	0.003
Cardioversion	24.1% (317)	23.9% (88)	24.1% (229)	0.942
Postablation sinus rhythm	96.8% (1275)	97.4% (359)	96.5% (916)	0.894

*Note*: Continuous variables are expressed as mean ± SD. Categorical variables are expressed as no. (%). Acute success rate was defined as ratio between the number of effectively isolated pulmonary veins (PVs) and the number of target PVs.

### 3.3 Safety

In total, 29/1318 (2.2%) patients had at least one procedural complication, equally distributed between the groups (7/368 [1.9%] for YOUNG vs. 22/950 [2.3%] for MIDDLE‐AGED, *p* = 0.646), as reported in Table 3. The most common complication was phrenic nerve paralysis, that was transient in 15 patients (1.1%) and permanent in one (0.1%) with no statistical difference between groups. Vascular complications were observed in seven patients (0.6%), and two patients experienced cardiac tamponade (0.2%). Cardiac tamponade was managed by immediate pericardiocentesis and interruption of the procedure; no deaths occurred. No statistical difference was observed in the incidence of complications between the two groups. No cerebrovascular complication (TIA/stroke), death, PV stenosis, hemothorax, or pericardial effusion were observed.

**Table 3:** Complications

Complications	Total (*n* = 1318)	≤45 years (*n* = 368)	60–65 years (*n* = 950)	*p*‐Value
Patients with at least one complication	2.2% (29)	1.9% (7)	2.3% (22)	0.646
Permanent diaphragmatic paralysis	0.1% (1)	0.0% (0)	0.1% (1)	1.000
Transient diaphragmatic paralysis	1.1% (15)	1.6% (6)	0.9% (9)	0.383
Atrial fibrillation (AF) fistula	0.1% (1)	0.0% (0)	0.1% (1)	1.000
Cardiac tamponade	0.2% (2)	0.0% (0)	0.2% (2)	1.000
Femoral pseudo‐aneurism	0.2% (2)	0.0% (0)	0.2% (2)	1.000
Aematoma	0.3% (4)	0.0% (0)	0.4% (4)	0.581
Other complication	0.3% (4)	0.3% (1)	0.3% (3)	1.000

### 3.4 Long‐term outcomes and predictors of AF recurrence

**Figure 1:**
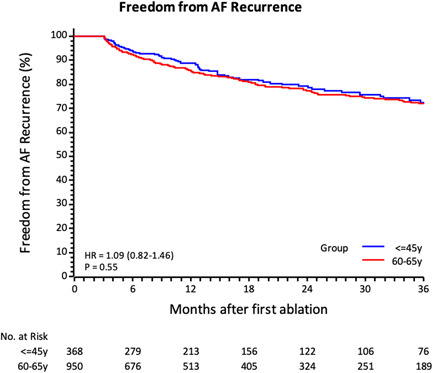
Kaplan–Meier estimate for atrial fibrillation (AF) recurrence in the two groups

The mean length of follow‐up was 23.6 ± 20.2 months. During follow‐up, 254/1318 (19%) patients had AF recurrence: 69/368 (18.7%) in the YOUNG and 185/950 (19.5%) in the MIDDLE‐AGED. The incidence rate for 100 patient‐years was 9.29 (95% CI: 7.72–11.18) in the YOUNG and 9.99 (95% CI: 8.93–11.19) in the MIDDLE‐AGED. The atrial arrhythmia recurrence rate was not statistically different between the two groups (*p* = 0.55). The time‐to‐first AF recurrence is shown in Figure 1. At the 12‐month follow‐up, the observed freedom from AF recurrence was 88.9% (95% CI: 84.7–92.0) in the YOUNG and 85.6% (95% CI: 82.9–88.0) in the MIDDLE‐AGED. At 36‐month follow‐up, freedom from AF was 72.4% (95% CI: 65.5–78.2) in the YOUNG and 71.8% (95% CI: 67.7–75.6) in the MIDDLE‐AGED, with no significant difference among groups (*p* = 0.550). Limited to paroxysmal AF, at 36‐months follow‐up freedom from AF recurrence was 74.6% (95% CI: 67.1%–80.6%) in the YOUNG and 72.6% (67.7%–76.8%) in the MIDDLE‐AGED with no significant difference (*p* = 0.34). In persistent AF, at 36‐months follow‐up freedom from AF recurrence was 57.5% (95% CI: 37.3%–73.3%) and 69.6% (95% CI: 61.2%–76.6%) in YOUNG and MIDDLE‐AGED patients respectively, with no significant difference (*p* = 0.14). However, the rate of repeat ablations following the index procedure (5.12% vs. 3.73%, *p* = 0.012) differs between YOUNG and MIDDLE‐AGED patients (Incidence Ratio 0.73, 95% CI: 0.57–0.93, *p* = 0.012). Several conditions possibly associated with AF recurrence were investigated by means of univariate and multivariate analyses in the YOUNG (Table 4). The Cox regression analysis identified that type of AF, use of antiarrhythmic drug, and beta‐blockers were predictors of atrial arrhythmia recurrence. At the multivariate analysis, only persistent AF (HR 2.45 [1.12–5.37], *p* = 0.025) resulted as independent predictor of AF recurrence. Echocardiographic parameters were not included in the multivariate model, due to correlation coefficient >0.25 or due to low number of patients with information recorded.

**Table 4:** Cox regression—atrial fibrillation (AF) recurrence in young patients

	Univariate analysis		Multivariate analysis	
Variable	Hazard ratio (HR)	*p*‐Value	HR	*p*‐Value
Gender (male)	0.87 (0.41−1.85)	0.721	0.62 (0.28−1.36)	0.236
BMI (continuous)	1.04 (0.98−1.11)	0.223		
BMI > 30	1.26 (0.55−2.86)	0.584		
Type of atrial fibrillation—paroxysmal	0.35 (0.18−0.66)	0.001		
Type of atrial fibrillation—persistent	2.86 (1.51−5.44)	0.001	**2.45 (1.12−5.37)**	**0.025**
Months from first atrial arrh. Episode (continuous)	1.00 (0.99−1.01)	0.906		
History of stroke/TIA	0.91 (0.13−6.60)	0.926		
Hypertension	0.53 (0.21−1.34)	0.180		
CHA₂DS₂‐VASc (continuous)^a^	0.84 (0.51−1.39)	0.503		
Diabetes	0.95 (0.13−6.86)	0.957		
Cardiomyopathy	1.15 (0.82−1.62)	0.422		
AAD	1.83 (1.00−3.36)	0.051		
Beta‐blockers	2.77 (1.53−5.01)	<0.001		
Ace‐inhibitors	0.67 (0.29−1.56)	0.352		
Diuretics	1.16 (0.28−4.78)	0.838		
LVEF (%) (continuous)	0.98 (0.94−1.01)	0.140		
Left atrial diameter (mm) (continuous)	1.02 (0.97−1.07)	0.513		

Abbreviations: BMI, body mass index; CKD, chronic kidney disease; LVEF, left ventricular ejection fraction; TIA, transient ischemic attack.

^a^ CHA2DS2‐VASc score risk ranges from 0 to 9, with higher scores indicating a greater risk of stroke.

## 4 DISCUSSION

This retrospective, real‐world project analyzed the results of CB‐PVI for the treatment of AF in a large cohort of patients aged ≤45. The data demonstrate that between young and middle‐aged patients there was no difference in critical parameters of safety and efficacy, including: (1) in the acute success rate of CB‐PVI; (2) in the rate of AF relapse after index procedure. However, the rate of repeat ablations following the index procedure was higher in young as compared to middle‐age. Persistent AF was the only predictor of AF recurrence in the cohort of patients aged <45.

### 4.1 Efficacy

To date, this is the first multicenter analysis to evaluate the differences in efficacy and safety of a single ablation technique (CB‐PVI) between young and middle‐aged patients. In our experience, CB‐PVI was not more effective in young patients than in middle‐aged patients. The analysis demonstrated the same rate of acute success, freedom from recurrent arrhythmia, in both groups, despite (as expected) older patients having more comorbidities (e.g., hypertension, diabetes mellitus, and chronic kidney disease), larger LA diameter and volume, and more persistent form of AF. The long‐term efficacy of CB‐PVI observed in our analysis (freedom from AF recurrence is 88.9% at the 12‐month follow‐up and 72.4% at the 36‐month follow‐up) overlaps that of Moran et al., who reported a freedom from AF relapse of 88% after a median follow‐up of 18 ± 10 months in a young cohort of patients <40 years.^8^ In other series including RF‐ and CB‐PVI, freedom from AF recurrence after a single procedure varied from 60% to 85% after 12 to 36 months of follow‐up.^6^, ^7^, ^10^, ^11^ Our incidence rate of AF relapse of 9.29 for 100 patient‐years, overlaps the results of Saguner et al., who reported a 5‐year arrythmia‐free survival of 44% in a very young cohort of 85 patients (age < 35 years).^9^

The same homogeneity of results was not maintained when efficacy of PVI is compared between young and older patients. Both Chun et al. and Tijskens et al. reported a significant lower recurrence rate after a single procedure in a population ≤45 years in comparison to a cohort of patients >45 years.^7^, ^11^ The heterogeneity of the techniques used for PVI (RF and CB) could explain the difference with our findings. Specifically, the reproducibility of CB‐PVI in patients with or without structural heart disease probably enhanced the effect of PVI in older patients.^14^ Very recently, in a larger single‐center experience, Hartl et al. showed that freedom from arrhythmia recurrence in a cohort of 786 patients treated with CB‐PVI was independent of age at 18 months and that age did not have an impact on procedural parameters and safety profile,^15^ confirming the results found by Sciarra et al.^16^ on 2534 AF patients.

### 4.2 Safety

Safety outcomes of the procedure did not differ between young and middle‐aged patients, with a very low complication rate overall in both cohorts. Transient phrenic nerve paralysis (1.2%) was lower than that reported in recent large studies with the second‐generation CB (Ciconte et al. reporting 8% and Aryana et al. reporting 7.6%),^17^, ^18^ and only one patient suffered from an unresolved phrenic nerve palsy during the study follow‐up period. Overall, our observations support previous data about the safety of PVI in young patients.^7^, ^8^, ^9^, ^10^, ^11^

### 4.3 Predictors of AF recurrence

In our analysis, persistent AF was independently associated with AF recurrence after CB‐PVI in young patients. Persistent AF was the most frequently observed independent predictor of AF recurrence both in the young and older cohort, and the other predictors observed were structural heart disease,^9^, ^11^ obesity, and LA diameter.^15^ The role of persistent AF as a predictor of arrhythmia recurrence may be explained (in part) by the advancement of atrial disease and the subsequent physiological atrial remodeling due to longer‐standing arrhythmia. Analysis limited to persistent AF shows a trend toward a greater recurrence rate in young people: these results could be explained by the possible role of extrapulmonary triggers observed in persistent AF in young people. Indeed, patients with persistent AF typically presents with additional non‐PV triggers, including: the PV antrum, posterior wall of the LA, superior vena cava, crista terminalis, coronary sinus, ligament of Marshall, and LA appendage. However, CB (unlike point‐by‐point RF ablation) is a method designed to achieve PVI only (without acting on different focal triggers); nevertheless, large multicenter randomized trials did not demonstrate any benefit for more complex ablation procedures.^19^ Consequently, a CB‐PVI remains a viable strategy in the treatment of patients with persistent AF.

### 4.4 Management of AF in young patients

Management of AF in young patients is challenging. Understanding the longer‐term effects of AF in young patients is vital as they will live with the disease ramifications for many decades. Current guidelines identify young symptomatic patients with paroxysmal AF and no structural heart disease as ideal candidates for catheter ablation.^3^ Previous studies showed better outcome in younger patients than in older ones.^7^, ^11^ In young patients, PV isolation leads to the elimination of AF trigger(s) before the development of atrial electrical and structural remodeling which can lead to the development of new atrial triggers and the progression of AF disease. Surprisingly, in our analysis, younger patients did not experience a better outcome as compared to a recurrence rate of almost 10% per year of follow‐up in the general AF patient population.^20^ Consequently, the history of patients who begin to suffer from AF very early will likely be characterized by several catheter ablations to control a multi‐factorial AF arrhythmia through the lifetime of disease.

## 5 STUDY LIMITATIONS

This analysis was based on a group of patients who underwent an AF catheter ablation with a specific technology and strategy (CB‐PVI), without predefined inclusion criteria. Due to the observational nature of the study, potential selection bias cannot be excluded. Although cryoablation technology is highly standardized, many procedural aspects are left to the discretion of the various centers, and thus, these factors may have influenced parameters of efficacy and safety. Moreover, the large number of patients and centers may have mitigated possible bias while demonstrating a real‐world clinical experience. We did not perform an electrophysiologic study in young patients before PVI, and this choice could have allowed us to identify a supraventricular tachycardia as the substrate of AF in around a quarter of young adult patients.^10^ Detection of AF recurrence was mostly based on symptoms; therefore, the efficacy of CB‐PVI might be overestimated in this population due to the lack of identification of asymptomatic episodes. The management of antiarrhythmic drugs during the blanking period and in the follow‐up was left to the individual centers: therefore, it is not possible to establish the role of antiarrhythmic therapy in preventing recurrences in the two groups. Finally, the patient follow‐up was left to the clinical practice at each center, and to mitigate the non‐standardized follow‐up, the patients were requested to share every documentation regarding ECGs, Holter monitors, hospital admissions, and/or in‐office cardiac visits.

## 6 CONCLUSIONS

In this large observational cohort, CB‐PVI for AF in young patients was not more effective than in older patients. The younger age did affect acute procedural results nor longer‐term AF recurrence after a single procedure. Importantly, procedures were safe in the young and older patients. Further studies are required to investigate very long‐term outcomes in young patients treated with CB‐PVI.

## Data Availability

The data that support the findings of this study are available from the corresponding author upon reasonable request.
